# Inter- and intraobserver variation between radiologists in the detection of abnormal parenchymal lung changes on high-resolution computed tomography

**DOI:** 10.4103/0256-4947.60518

**Published:** 2010

**Authors:** Hanaa Al-Khawari, Reji P. Athyal, Osama Al-Saeed, Prio N. Sada, Sana Al-Muthairi, Adel Al-Awadhi

**Affiliations:** a From the Department of Clinical Radiology, Faculty of Medicine, Kuwait University, Kuwait; b From the Department of Clinical Radiology, Al Amiri Hospital, Kuwait University, Kuwait; c From the Department of Medicine, Faculty of Medicine, Kuwait University, Kuwait

## Abstract

**BACKGROUND AND OBJECTIVES:**

Radiological and histological evaluations are affected by subjective interpretation. This study determined the level of inter- and intraobserver variation among radiologists for detection of abnormal parenchymal lung changes on high resolution computed tomography (HRCT).

**METHODS:**

HRCT images of 65 patients known to have systemic lupus erythematosus (with clinical pulmonary involvement) were retrospectively reviewed by four nonthoracic radiologists (two with expertise in magnetic resonance [MR] and two general radiologists). Each radiologist read the scans twice, with an interval between readings of at least 6 months. The interobserver variation among the first and second readings of the four radiologists and the intraobserver variation of each radiologist's two readings were assessed by the kappa statistic.

**RESULTS:**

There was good agreement between the first and second readings of each radiologist. There was moderate agreement between the two readings of one MR radiologist (kappa=0.482); the other three radiologists had kappa values that were good to excellent (0.716, 0.691, and 0.829). There was a clinically acceptable level of interobserver variability between all radiologists. The agreement was fair to moderate between the MR radiologist and the other observers (kappa range: 0.362-0.519) and moderate to good between the other three radiologists (0.508-0.730).

**CONCLUSION:**

The interpretation of imaging findings of abnormal parenchymal lung changes on HRCT is reproducible and the agreement between general radiologists is clinically acceptable. There is reduced agreement when the radiologist is not involved on a regular basis with thoracic imaging. Difficult or indeterminate cases may benefit from review by a chest radiologist.

High-resolution computed tomography (HRCT) of the lungs is being increasingly used in the diagnosis and treatment of diffuse parenchymal lung disease (DPLD). Until recently, open lung biopsy was the most reliable guide to the likely outcome, with histological appearance suggesting inflammation indicating a relatively good prognosis and fibrotic change implying a poor outcome.^1^–^3^ However, the invasive nature of open lung biopsy is a serious drawback. HRCT of the lungs is now being recognized as an excellent noninvasive technique for providing prognostic information and open lung biopsy is being relegated to situations where the findings on HCRT are equivocal. Noninvasive investigations such as pulmonary function tests, bronchoalveolar lavage, and chest radiography do not consistently identify reversible disease.^4^

The emergence of HRCT as a versatile diagnostic test is due to its diagnostic accuracy. Radiological and histological evaluations are affected by subjective interpretation and this observer variation can affect the reproducibility of a diagnostic test. With the increasing popularity and accessibility of HRCT, the majority of patients with interstitial lung disease are currently managed on the basis of HRCT observations, without histological evaluation. Thus, knowledge of observer variation in the interpretation of HRCT is needed.^5^ We determined the degree of inter- and intraobserver variation in the detection of abnormal parenchymal lung changes on HRCT by quantifying the extent to which radiologists in a general hospital agree with each other with regard to HRCT findings.

## METHODS

The HRCT images of 65 patients known to have systemic lupus erythematosus (SLE) and treated at the chest clinic in a general hospital were retrospectively reviewed by four radiologists. The four radiologists included two magnetic resonance radiologists (HK and OS) and two general radiologists (RA and PS). These participating radiologists, all working at a teaching hospital, had completed their general radiological training 8-16 years earlier at different institutions. The two MR radiologists had received special training in MRI for 1-2 years and had been mainly involved in MRI reporting for the past 4-6 years. All four radiologists were aware that the patients in the study had SLE, but no additional clinical information related to the respiratory system was made available to them when reviewing the HRCT images. Each radiologist read the scans twice, with an interval of at least 6 months between the two readings.

The radiologists determined whether the following radiological lung features of SLE were present: diffuse thickening, nodular thickening, alveolar honeycombing, perivascular nodularity, peribronchial nodularity, calcification, bronchiectasis, scarring, a ground-glass appearance, emphysematous bullae, prominent vasculature, pleural calcification, diffuse pleural thickening, nodular thickening with small and large nodules. After assessment, the results were recorded as normal lung parenchyma or abnormal lung parenchyma (if there were any parenchymal lung changes). The findings were described based on the location of the abnormalities (in the upper lobe, middle lobe (or lingular segments on the left), and lower lobe) and also according to the central or peripheral (outer third of the lung) nature of the abnormalities. The interobserver variation between the first and second readings of the four radiologists and the intraobserver variation between each radiologist's two readings were identified and the results assessed by the kappa statistic.

The images were obtained on a CT scanner (Somatom 4 Plus Spiral CT, Siemens, Germany), with 1-mm collimation at full inspiration. Scans were obtained at 10-mm intervals from the apices to the bases, with the patient in the supine position. The images were reconstructed with a high-spatial-frequency algorithm and photographed at window settings appropriate for viewing the lung parenchyma. All the images were evaluated on hard copy.

**Figure 1:**
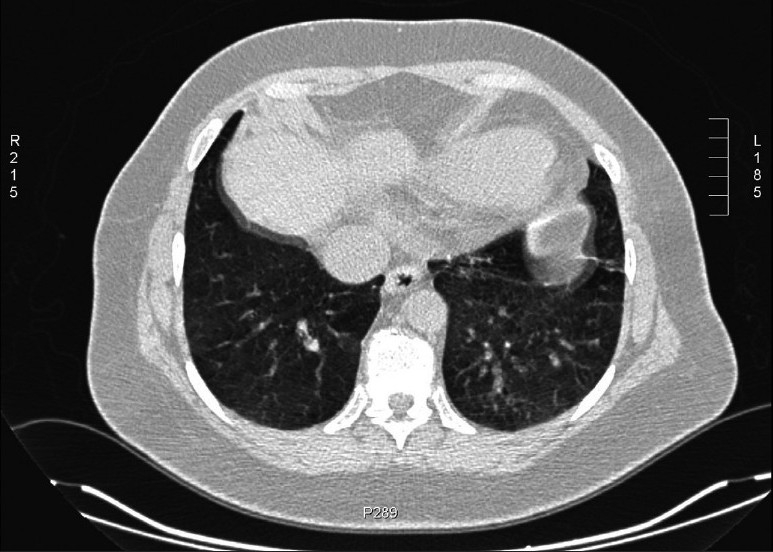
The image illustrating different observers' opinions evaluated as ground-glass appearance against improper breath-hold during scanning. Image used to evaluate observer variablity on ground-glass appearance vs. breath-holding during scanning.

**Figure 2:**
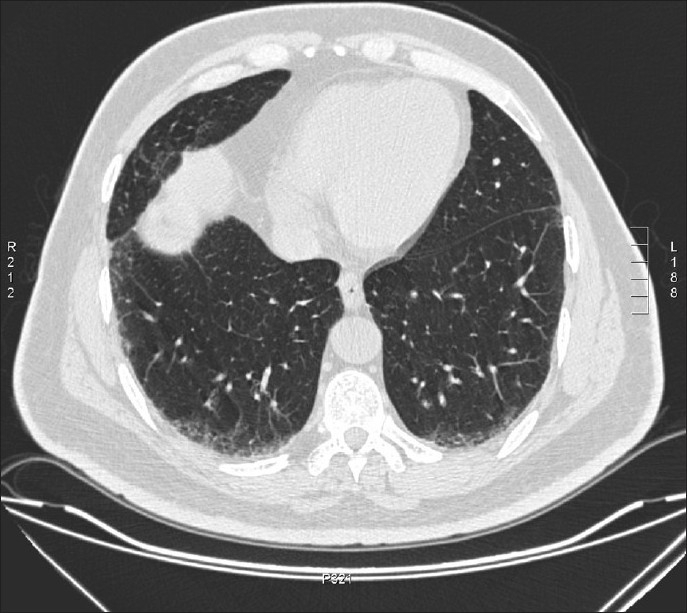
The image illustrating different observers' opinions as interstitial parenchymal change against dependent edema.

Interobserver and intraobserver variability in grading appearances on HRCT were quantified (Figures 1 and 2) using the kappa statistic, which measures agreement between observers while accounting for chance.^6^ The data trends were evaluated by analysis of variance or the chi-square test. All statistical analyses were performed using STATA data analysis software (Computing Resource Center, Santa Monica, CA, USA). Observer agreement was categorized by kappa values as poor (<0.20), fair (0.20-0.39), moderate (0.40-0.59), good (0.60-0.79), or excellent (>0.80).^7^

## RESULTS

The first and second readings (intraobserver variability) for each radiologist were compared (Table 1). There was statistically significant agreement between the first and second readings among all four radiologists. There was moderate agreement between the two readings of one of the MR radiologists (HK) with a kappa value of 0.482; the other three radiologists had kappa values of agreement that were good to excellent (0.716, 0.691, and 0.829). The *P* value of all four radiologists was.001 which indicates there is significant agreement between the observations of all the observers.

**Table 1:** Agreement between the two readings for each radiologist (intraobserver variability).

Radiologist	Agreement on normal	Agreement on abnormal	Disagreement between the two readings	Kappa value*	*P* value
HK	18/65	26/65	21/65	0.482	.001
OS	19/65	32/65	14/65	0.716	.001
PS	29/65	21/65	15/65	0.691	.001
RA	24/65	30/65	11/65	0.829	.001

* a value of 1 indicates perfect agreement

Agreement between radiologists (interobserver variability) was also compared for the two readings (Table 2). There was statistically significant interobserver agreement between all radiologists. This was fair to moderate between the MR radiologist (HK) and the other observers, with the kappa value ranging between 0.362 to 0.519. Among the other three radiologists the interobserver variability ranged from moderate to good (0.508 to 0.730).

**Table 2:** Agreement between the radiologists (interobserver variability) for readings I and II.

Reading I	HK	OS	PS
OS	0.362		
PS	0.462	0.666	
RA	0.451	0.730	0.730

**Reading II**	**HK**	**OS**	**PS**

OS	0.551		
PS	0.339	0.508	
RA	0.519	0.620	0.604

## DISCUSSION

Observer variability has been a problem ever since imaging began to contribute to the diagnosis of diffuse parenchymal lung disease, beginning as far back as the 1940s.^8^,^9^ On chest radiography, the inability of the observer to compensate for under- and overexposure of films, varying classification systems, and a lack of familiarity with radiological manifestations were some of the problems faced. Further, previous studies assessing the role of chest radiography in fibrosing alveolitis have shown little correlation between appearances on chest radiography and histological findings.^10^ These factors have resulted in an increasing reliance on computed tomography. With the widespread availability of HRCT scans for diagnosing diffuse lung disease, treatment is often initiated based on the interpretation of imaging findings, without recourse to histological confirmation. The aim of our study was to quantify the degree of intra- and interobserver variability between nonthoracic radiologists in the evaluation of HRCT images in patients with SLE attending a chest clinic in a general hospital. We focused on nonthoracic radiologists as we felt it would be more appropriate to assess observer variation among this group of radiologists who by and large report HRCT scans without access to dedicated chest radiologists and since they are increasingly providing the opinions on which decisions are made.

We used the kappa coefficient of agreement to evaluate observer variability as this accounts for chance agreement. The clinical significance of a kappa value depends upon its context and the values cannot be always compared between studies as it is dependent on disease prevalence.^11^ In a recent review of studies that have used the kappa coefficient, the authors concluded that a value greater than 0.4 could be considered an acceptable level of observer variability.^7^ We have followed the classification system used by Coblentz et al., where agreement is quantified as poor, fair, moderate, good, or excellent.^7^ The second reading in our study could potentially be affected by the training effect and bias in the detection of findings as a consequence of the first reading. To minimize this, we ensured that there was a gap of at least 6 months between the two readings. The interobserver agreements on the two sets of readings showed similar trends and both sets were included in the analysis.

A few of the early studies performed to evaluate the diagnostic accuracy of HRCT also incorporated an evaluation of observer variation. However, these studies included many unusual diagnoses^12^,^13^ and the number of observers were small.^13^–^15^ Grenier et al.^12^ and Lee et al.^14^ reported fairly high kappa values of 0.78 and 0.75 for the diagnosis of diffuse interstitial lung disease. The kappa value is highly dependent on disease prevalence and thus studies may not be strictly comparable; nonetheless, these are higher values than were seen in our study and other studies in the literature. Collins et al.^10^ assessed observer variation in diagnosing pattern type and disease extent in fibrosing alveolitis on HRCT scans and reported an interobserver variability on CT scan that was clinically acceptable. They demonstrated a higher level of confidence and less observer variability for CT scans than for chest radiography. Jokhoh et al^16^ assessed the diagnostic accuracy of HRCT in idiopathic interstitial pneumonias and obtained a kappa value of 0.55. The differential diagnosis was however limited to just five types of idiopathic interstitial pneumonias, which probably increased the kappa value. In another study^17^ of patients with suspected idiopathic pulmonary fibrosis (IPF) in which the need for lung biopsy was assessed, the agreement between radiologists regarding the presence or absence of IPF was 0.54 and 0.50, respectively. Thomeer et al.^18^ evaluated the interobserver variation and accuracy of the diagnosis of IPF by respiratory physicians across six European countries who were given 179 HRCT scans for evaluation. They found the interobserver agreement to be fair to moderate between readers. The overall accuracy of the clinical diagnosis was good (87.2%). Our study is slightly different in that we specifically studied patients with SLE to evaluate the agreement between radiologists in the detection of findings. Radiopathological correlation was not performed as the objective was only to ascertain the degree of agreement in the evaluation of imaging. Our results are however similar with regard to the interobserver variation between radiologists in that the agreement between the observers (kappa value) ranged from fair to good. In our study, the intraobserver agreement ranged from moderate to excellent (0.482 to 0.829), indicating that the detection of relevant findings and interpretation of imaging appearances are reproducible. The interobserver agreement between one of the MR radiologists and the other three observers, was relatively poor, but it was still fair to moderate (the kappa value ranging from 0.362 to 0.519). This is no doubt due to the fact that this MR radiologist was not exposed on a daily basis to chest imaging. Nonetheless, the degree of interobserver variation is still comparable to that in other studies and shows that there is a reasonably good level of agreement between radiologists. The interobserver variability between the other three radiologists was even better, ranging from moderate to good (kappa: 0.508 to 0.730). We did not use a reference or gold standard in this study as the attempt was not to perform a radiological-pathological correlative analysis or to compare the radiological impression with a final proven diagnosis. Thus, a high level of agreement between observers does not necessarily indicate a high level accuracy of diagnosis.

The role, if any, of the better quality images produced by the newer and more versatile CT machines in the reduction of inter- and intraobserver variability has not been specifically studied, although this might be of some significance.

Significant variability between experienced histopathologists has been documented in the semiquantitative grading of interstitial fibrosis, intra-alveolar inflammation, and interstitial inflammation in open-lung biopsy samples.^19^ The kappa values in all these instances were less than 0.30. In a study of diffuse lung disease by Nicholson et al.,^20^ where pathologists could choose a diagnosis from a specified list of 15 categories, the kappa values for agreement between pathologists in tertiary referral cases was found to be 0.38. In comparison, the agreement among radiologists was found to be 0.34 for tertiary referral cases in the study by Aziz et al.^5^

Although we did not classify our patients based on the referral pattern from peripheral clinics, there would no doubt be a lower degree of agreement in tertiary referral cases. This highlights the fact that in difficult cases of DPLD it will not be sufficient to rely on either imaging or pathology in isolation. It is important in these situations to integrate the clinical information with HRCT and pathology findings (if available) before initiating treatment or formulating a final diagnosis.

In conclusion, our study quantifies the level of inter- and intraobserver variability in the detection of abnormal parenchymal lung changes of SLE. The interpretation of imaging findings is reproducible and comparable. The agreement between general radiologists is clinically acceptable. There is some variability in interpretation and reduced agreement between one MR radiologist (who was involved in thoracic CT imaging only infrequently) and the others. Difficult or indeterminate cases may benefit from review by a dedicated thoracic radiologist and, in some situations, a lung biopsy.
